# Plasmid-Borne Biosynthetic Gene Clusters within a Permanently Stratified Marine Water Column

**DOI:** 10.3390/microorganisms12050929

**Published:** 2024-05-02

**Authors:** Paraskevi Mara, David Geller-McGrath, Elizabeth Suter, Gordon T. Taylor, Maria G. Pachiadaki, Virginia P. Edgcomb

**Affiliations:** 1 Geology & Geophysics Department, Woods Hole Oceanographic Institution, Woods Hole, MA 02543, USA; pmara@whoi.edu; 2 Biology Department, Woods Hole Oceanographic Institution, Woods Hole, MA 02543, USA; mcgrath1@mit.edu (D.G.-M.); mpachiadaki@whoi.edu (M.G.P.); 3 Biology, Chemistry and Environmental Science Department, Molloy University, New York, NY 11570, USA; esuter@molloy.edu; 4 School of Marine, Atmospheric and Sustainability Sciences, Stony Brook University, New York, NY 11794, USA; gordon.taylor@stonybook.edu

**Keywords:** plasmids, secondary metabolites, BGCs, metagenome assembled genomes, MAGs, oxygen-depleted water columns, Cariaco Basin

## Abstract

Plasmids are mobile genetic elements known to carry secondary metabolic genes that affect the fitness and survival of microbes in the environment. Well-studied cases of plasmid-encoded secondary metabolic genes in marine habitats include toxin/antitoxin and antibiotic biosynthesis/resistance genes. Here, we examine metagenome-assembled genomes (MAGs) from the permanently-stratified water column of the Cariaco Basin for integrated plasmids that encode biosynthetic gene clusters of secondary metabolites (smBGCs). We identify 16 plasmid-borne smBGCs in MAGs associated primarily with Planctomycetota and Pseudomonadota that encode terpene-synthesizing genes, and genes for production of ribosomal and non-ribosomal peptides. These identified genes encode for secondary metabolites that are mainly antimicrobial agents, and hence, their uptake via plasmids may increase the competitive advantage of those host taxa that acquire them. The ecological and evolutionary significance of smBGCs carried by prokaryotes in oxygen-depleted water columns is yet to be fully elucidated.

## 1. Introduction

Genes can be transferred between bacteria by three main mechanisms: transformation, where extracellular DNA is taken up by a recipient cell; transduction, where genetic material is transferred via bacteriophages; and conjugation, where genetic material is transferred between cells via a conjugal pilus. Plasmids are mobile elements that carry genes typically related to antibiotics or other secondary metabolites and they are exchanged between microorganisms during conjugation or can be acquired via transformation. Using marine sediments and mesocosm experiments, transfer of mobile DNA through plasmids was demonstrated using a naturally occurring plasmid and a mixed marine bacterial community [1,2]. While studies of terrestrial environments have revealed the importance of secondary metabolite biosynthetic gene clusters (smBGCs), we are just starting to learn about the roles they may play within marine prokaryotic communities, particularly in oxygen-depleted water columns (ODWCs: O_2_ ≤ 20 μmol kg^−1^). As an example, siderophores that facilitate iron uptake and occur as smBGCs, were found encoded in plasmids of *Rhizobium* sp. that inhabit the rhizosphere of terrestrial plants [3].

Paoli et al. (2022) [4] investigated the biosynthetic potential of microorganisms in the open ocean by combining analyses of ~10,000 microbial genomes from cultured isolates and single cell amplification with ~25,000 new draft genomes from >1000 seawater samples from diverse global epipelagic, mesopelagic, and bathypelagic zones. This analysis revealed ~40,000 putative and mostly new biosynthetic gene clusters, and highlighted that certain uncultured bacterial lineages have high abundances of smBGCs (e.g., ‘*Candidatus* Eudoremicrobiaceae’). In addition, metagenome surveys of cultured environmental bacterial isolates from marine sediments and the surface ocean showed that bacteria from these habitats commonly acquire and integrate mobile smBGCs into their genomes, a trait that can enhance their fitness and enable more cosmopolitan distributions (e.g., [5,6,7,8]).

**Figure 1:**
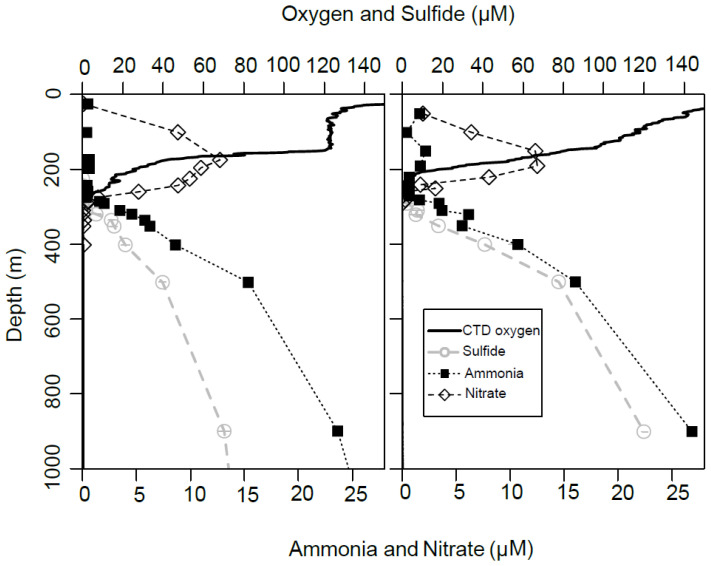
Depth profiles of water column biogeochemistry during May (**left**) and November (**right**) 2014.

While previous research indicates the importance of smBGCs under fully oxic conditions, the role and importance of smBGCs carried on plasmids under O_2_ depleted/anoxic conditions in marine water columns has not been examined. The Cariaco Basin, Venezuela, is an end member in the continuum of ODWCs, with permanently anoxic (zero O_2_), and euxinic (sulfidic) conditions below ~250 and ~275 m, respectively ([9]; Figure 1). This water column is characterized by steep oxygen and redox potential gradients (=redoxcline). Along the Cariaco redoxcline diverse particle-associated (PA: >2.7 µm) and free-living (FL: 0.22 to 2.7 µm) microbial communities exist that have varying energy and oxidant demands [10,11]. Previous analyses of metagenomic-assembled genomes (MAGs) and metatranscriptomes along the Cariaco’s redoxcline provided evidence that FL and PA microbial communities encode biosynthetic gene clusters associated with various secondary metabolites [12].

The goal of this study was to learn whether smBGCs are carried on plasmids in low oxygen marine systems, what types of smBGCs are encoded, and by which taxa. To address this, we investigated 565 MAGs affiliated with particle-associated and free-living prokaryotic communities recovered from water samples along the Cariaco Basin redoxcline.

## 2. Materials and Methods

### 2.1. Sample Collection, Extraction and Sequencing of DNA and RNA Samples

Water samples for DNA and RNA were collected from 6 depths during two cruises in May and November 2014 in Cariaco Basin, Venezuela. Depths sampled during those cruises spanned the redoxcline of Cariaco Basin and included three sampling depths along the oxycline (O_2_: ~80 μM to 0.4; Depths: 103, 196, 235 m in May, and 148, 200, 237 m in November), two anoxic water depths at 295 m and 315 m in May, and 247 m and 267 m in November, and a euxinic water depth (zero O_2_ and >80 µM H_2_S) sampled from 900 m [10,12]. For DNA samples, 8–10 L water samples were gravity-filtered sequentially through EMD Millipore 2.7 µm glass fiber membranes 47 mm diameter (PA fraction), and then through 0.2 µm Sterivex filters (FL fraction), then preserved in lysis buffer. For RNA samples, 2–3 L were collected sequentially through EMD Millipore 2.7 μm glass fibre filters (for PA), then through 0.2 μm Millipore polysulfone membranes (for FL) using the Microbial Sampler- In Situ Incubation Device (MS-SID) and preserved in RNAlater [12,13,14,15]. All filters were stored frozen onboard at −20 °C and moved to −80 °C at the lab until further processing. DNA was extracted with cell lysis (lysozyme and proteinase K treatments) and phenol/chloroform as described in [10]. Extracted DNA was sent to Georgia Genomics and Bioinformatics Core for library preparation and PairedEnd 2 × 150 bp Illumina NextSeq sequencing and processed as described in [12]. Protocols for RNA extractions and sequencing cDNA libraries are reported in [12].

### 2.2. MAG Co-Assembly, Binning, and Taxonomic Assignment

Reconstruction of MAGs was performed as described in [12]. The trimmed reads of metagenomic datasets from the anoxic and sulfidic depths (314 and 900 m in May, 267 and 900 m in November) and both PA and FL fractions were co-assembled into contigs using SPAdes 3.11.1 with default values and flag “–meta”. Assembled contigs were binned using MetaBAT 2.12.1 with default values. CheckM 1.0.1161 was used to estimate the completeness and contamination of the reconstructed genomes. Only MAGs with ≥75% complete and ≤5% contamination were used for the downstream analysis. The taxonomic placement of the MAGs was performed with GTDB-Tk85 2.1.1. The taxonomic identification of the recovered MAGs revealed the presence of three known lab contaminants, including *Burkholderia* contaminants that were removed from any downstream analysis.

### 2.3. Calculation of MAG Relative Abundances and Differential Abundance Analysis

A total of 565 MAGs were recovered from the oxycline, shallow anoxic, and euxinic layers of the Cariaco Basin. To dereplicate the MAGs, the Anvi’o 4 workflow was implemented [12]. Reads from 48 metagenomic samples (two sample types: PA- and FL-fractions, two replicates per fraction from six depths, taken over two sampling time points  =  48 metagenomes) were mapped to each MAG using the BWA 2.0 aligner via the CoverM 0.6.1 command line tool [12]. Differential abundance (PA-abundant, FL-abundant or no preference) was determined for individual MAGs exhibiting differences in abundance between sample type (PA, FL) and water layer (oxycline, shallow anoxic, euxinic). A count matrix was created with rows containing individual MAG read mapping counts and with metagenomic samples as columns. Count data for all MAGs were analyzed to calculate DESeq2 1.34.0 size factors for cross-sample count normalization. The differential abundance of MAGs between size fractions and water layers was modeled using the DESeq2 negative binomial model with the metadata variables of fraction size and water layer in which “count” was the dependent variable and “fraction” as well as “water layer” were independent variables. The significant differential abundances of MAGs (with an FDR-corrected *p* < 0.05) identified by comparing the PA and FL samples were grouped by water layer, and direct comparisons were made between normalized counts of significantly differentially abundant MAGs from the oxycline, shallow anoxic, and euxinic depths.

### 2.4. Scanning of MAGs for BGCs, Functionally Annotating Genes within Clusters, and Comparing Mined Clusters to the MiBIG Database BGCs

Genes encoded in the 565 MAGs were predicted using Prodigal 2.6.3 and the resulting genes of each MAG were individually scanned for smBGCs using antiSMASH 6.0 with default parameters as in [12]. The GUNC command line tool and manual curation were used to assess contigs from Cariaco MAGs for potential chimerism and/or contamination. Redundancy of the predicted biosynthetic cluster sequences recovered from the Cariaco MAGs was assessed using the BiG-SCAPE 1.1.4 command line tool with default parameters. Gene clusters with a total length less than 10 kb were discarded from downstream analysis to minimize the inclusion of fragmented smBGCs in our data (see also [12]). Genes predicted using Prodigal were scanned using the InterProScan 581 and Prokka 1.14.6 command line tools with default parameters for functional annotations, as well as during the implementation of the antiSMASH 6.0 pipeline with the antiSMASH HMM databases as in [12]. We manually searched the resulting annotations for genes and domains that encoded a variety of functions, such protein domains involved in post-translational modifications. Results comparing mined Cariaco smBGCs to the MiBIG database BGCs were scraped using R from the output HTML files from scanning each MAG with antiSMASH 6.0.

### 2.5. Identification of Putative Plasmids Encoding BGCs in the MAGs, and Metatranscriptomic Read Mapping of RNA-Seq Data to the Plasmids

All Cariaco Basin MAGs identified to contain BGCs using antiSMASH 6.0 were examined for encoding putative plasmids using PlasClass 0.1.1 [16] and PlasFlow 1.1.0 [17] tools with default settings. These tools can recover linear plasmid sequences from assembled metagenomes and can classify contigs in a metagenomic assembly that are of plasmid origin. Of the positive plasmid classifications, the contigs that contained one or more smBGC, and had a size of ≥10 kilobases and were classified as a plasmid by both tools were retained for downstream analyses. Visualizations of representative plasmids were constructed using SnapGene Viewer 7.0.2. Metagenomic and metatranscriptomic reads were mapped to MAG contigs using coverM (https://github.com/wwood/CoverM, accessed on 15 November 2022), including only alignments incorporating at least 50% of the length of a read pair with at least 95% identity. Alignment counts were normalized using the reads per kilobase million (RPKM) method. Average contig expression of MAG contigs not containing rRNA genes was calculated and compared to the expression of putative plasmid contigs across all samples using the RPKM-normalized mapping results.

### 2.6. Assesment of Plasmid and BGC Sequence Similarity, and Taxonomic Classification of Plasmids

The sequence similarity of the plasmid contigs and the smBGCs was assessed using Sourmash 4.5.0 [18] and CD-HIT 4.8.1 [19]. Sourmash “scaled” sizes of 20, 100, and 1000 were used with the “sourmash dna sketch” command, followed by the “sourmash compare” and “sourmash plot” commands. CD-HIT sequence identity thresholds of 60, 70, 80, and 90 specifieid with the “-c” flag were used with the “cd-hit” command. Additionally, the Contig Annotation Tool (CAT; [20]) was used to classify each of the plasmid contigs using default parameters with the GTDB CAT database.

## 3. Results

We analyzed 565 MAGs recovered from the Cariaco Basin [12], with gene annotation tools (KofamScan, InterProScan, BLASTP, DRAM; [21,22,23]) and methods designed to identify secondary metabolites (antiSMASH 6.0; [24]). With these analyses we found that 239 of the 565 MAGs contained one or more contigs encoding a smBGC along with genes typically associated with gene mobility and plasmid conjugation (requiring e-values < 1 × 10^−15^). These 239 MAGs were primarily affiliated with Pseudomonadota, Planctomycetota, Thermodesulfobacteriota, and Omnitrophota. While these findings indicate the presence of plasmid-transferred smBGCs in these MAGs, for accuracy we re-examined all MAGs encoding smBGCs with bioinformatic tools designed to more reliably predict plasmid sequences in genomes and metagenomes. PlasClass 0.1.1 [16] and PlasFlow 1.1.0 [17] recover linear plasmid sequences and contigs of plasmid origin from genomes and metagenomes using additional features for plasmid prediction (e.g., %GC content, *k*-mer frequency). Using these pipelines we identified 38 integrated plasmids (episomes; [25]) with probability scores > 0.5 that carried smBGCs (hereafter “smBGC episomes”). These 38 episomes were in 28 MAGs affiliated mainly with Planctomycetota and Pseudomonadota (14/28 MAGs; Supplementary Table S1).

We selected 14 MAGs to discuss in this paper based on the following criteria: (a) must have been identified by both PlasClass and PlasFlow tools, (b) needed to contain ≥1 smBGC episome that was more than 10 kilobases in size, and (c) needed to encode one or more genes indicative of the capacity for gene transfer. These genes include plasmid conjugation (*tra* and *trb* operons), pilus biogenesis and assembly (e.g., Flp/Tda proteins), and enzymes associated with plasmid partitioning (Par), transposition, integration, recombination (e.g., transposases, integrases, recombinases), with e-values stronger than 1 × 10^−15^. We acknowledge that our criteria may underestimate the actual numbers of smBGC episomes in the Cariaco Basin’s database. Nonetheless, we choose this approach because existing literature on mobile smBGCs in ODWCs is rare (if any), and therefore, we want to describe only those MAGs that confidently encode plasmid-borne smBGCs.

The 14 MAGs contained 16 episomes from 11 to 197 kbp in size, and were affiliated with 2 archaeal phyla (Altiarchaeota, Halobacterota) and 4 bacterial phyla (Planctomycetota, Pseudomonadota, Verrucomicrobiota, Myxococcota). Two Planctomycetota MAGs (CarAnox_MAG_1628 and CarAnox_MAG_406) each had two contigs with smBGC episomes (Table 1). Eleven MAGs encoding smBGC episomes were recovered from anoxic and euxinic depths, while three MAGs (two affiliated with Pseudomonadota and one with Planctomycota) were recovered from all examined water regimes (oxycline, shallow anoxic, euxinic; Table 1). To reveal if the identified smBGC episomes are expressed, we mapped metatranscriptome reads generated from the same examined depths [12] to the 16 smBGCs. The episomes that received transcript mapping were from anoxic and euxinic depths (Table 1). Undetected expression of genes on episomes in MAGs from other depths should be interpreted with caution.

**Figure 2:**
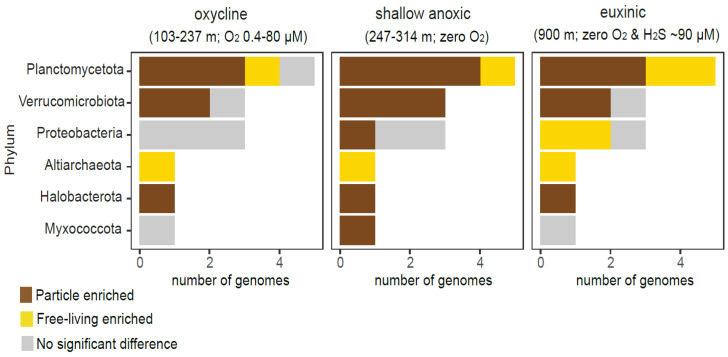
Preference of MAGs for particle-associated and free-living fractions along the oxycline (103–237 m), shallow anoxic (247–314 m) and euxinic regimes (900 m). “No significant difference” indicates adjusted *p* values > 0.05.

We observed that archaeal MAGs encoding smBGC episomes were enriched either in the FL fraction (Altriarchaeota) or PA fraction (Halobacterota) along the Cariaco Basin’s redoxcline (adjusted *p* < 0.05; Figure 2). For the bacterial MAGs encoding smBGC episomes we find that these bacterial phyla were either enriched in the PA fraction under all examined geochemical regimes (e.g., Verrucomicrobiota, Planctomycetota) or became enriched in the FL fraction only at certain depths (adjusted *p* < 0.05; Figure 2). As an example, all Pseudomonadota MAGs were equally abundant in the FL or PA fractions within the oxycline (adjusted *p* > 0.05), but the abundance of some Pseudomonadota MAGs was enriched in the FL faction at 900 m (euxinic regime).

AntiSMASH analyses indicated that the 16 smBGC episomes were associated primarily with post-translationally modified peptides (RiPPs; 7/16), terpene production (3/16) and non-ribosomal peptides (NRPs; 3/16) (Table 1). Within the transferred smBGCs, other genes associated with basic metabolic and cellular functions were also detected, and included transporters and symporters of minerals and organic ions (e.g., Cu^2+^, Na^+^/H^+^, Ni, As, ABC and MFS transporters), genes involved in carbohydrate metabolism and cell wall/peptidoglycan synthesis (e.g., mannose-1-phosphate guanylyltransferase, phosphomannomutase, MurNAc-6-P etherase), and in vitamin and amino acid biosynthesis (cob(I)alamin adenosyltransferase, thiamine-monophosphate kinase, histidine ammonia-lyase, asparagine synthase). Additional genes within the smBGC clusters included genes related to plasmid conjugation and transfer (e.g., *tra* genes and genes associated with the type IV secretion system) and cellular movement (e.g., flagellum synthesis). Detailed information on the function and gene position of all identified genes carried on the smBGC episomes can be found in Supplementary Table S2.

**Figure 3:**
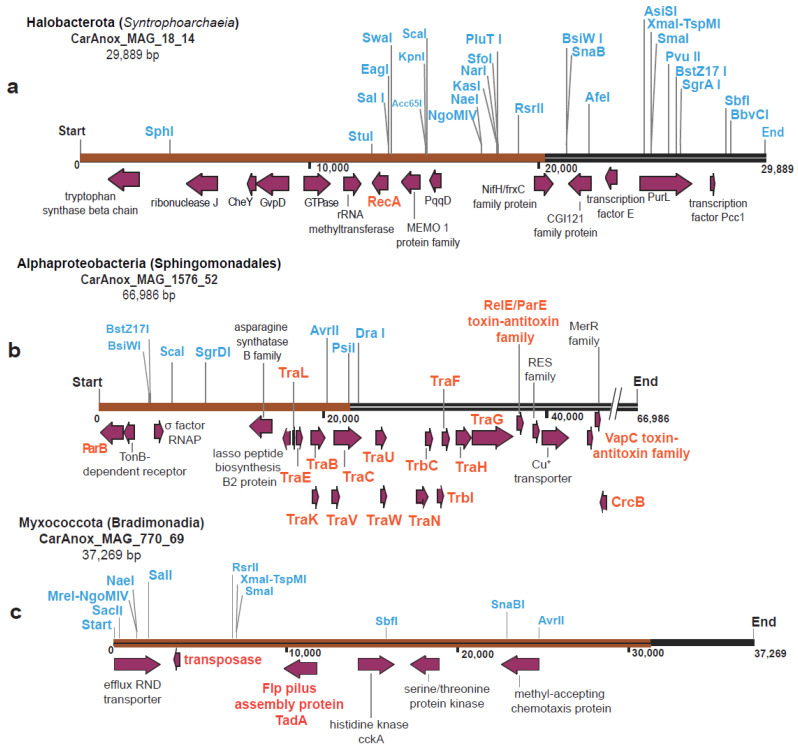
(**a**). Map of the representative archaeal episome identified in CarAnox_MAG_18 contig 14 that encoded RRE (RiPP) smBGC (brown color). (**b**). Map of the representative bacterial episome identified in CarAnox_MAG_1576 contig 52 that encoded RiPP smBGC (brown color). (**c**). Map of the representative bacterial episome identified in CarAnox_MAG_770 contig 69 that encoded NRP smBGC (brown color). Genes associated with plasmid conjugation, partitioning, recombination, pilus biogenesis, and toxin/antitoxin systems (indicators of plasmid mobility) are shown in orange. Enzyme restriction sites are shown in blue. The gene annotation for all genes encoded on the representative episomes shown in Figure 3a–c can be found in https://zenodo.org/records/10144545 (accessed on 22 July 2023).

To elaborate further into the gene content and gene position of the smBGC episomes, we selected one representative archaeal and two representative bacterial episomes, and constructed their maps (Figure 3). Specifically, the representative maps were constructed for (a) an archaeal MAG annotated to Syntrophoarchaeia encoding a RiPP recognition element (RRE) smBGC (CarAnox_MAG_18_14) (Figure 3a), (b) a bacterial MAG affiliated with Myxococcota encoding a RiPP smBGC (CarAnox_MAG_770_69) (Figure 3b), and (c) a bacterial MAG affiliated with Alphaproteobacteria encoding a NRP smBGC (CarAnox_MAG_1576_52) (Figure 3c). The Syntrophoarchaeia MAG was selected because it is one of two archaeal MAGs carrying smBGC episomes, and the one that received transcript mapping, suggesting the episome is expressed (Table 1). The MAG annotated to Alphaproteobacteria was selected because it encodes a suite of well-described genes typically involved in plasmid transfer (*tra* genes). Finally, the Myxococcota MAG was of interest because little is known about the ecological role and genomic structure of this taxon, particularly in ODWCs (e.g., [26,27,28]). It is postulated that Myxococcota produce a plethora of secondary metabolites that support their different ‘social’ traits in the marine environment (e.g., predation, sporulation, fruiting body formation; [29,30,31]). We also note that signatures of these selected representative taxa (Syntrophoarchaeia, Alphaproteobacteria, Myxococcota) are abundantly detected in the Cariaco Basin [10,12] and in similar, permanently anoxic pelagic systems (e.g., Black Sea; [32]). Aside from smBGCs and genes associated with plasmid conjugation/mobility, these maps also revealed episome-encoded genes for transporters, rRNA and tRNA modifications, cell signaling (e.g., kinases and proteins for cell division), transcription, nitrogen metabolism, and two-component regulatory systems involved in chemotaxis. In addition, they also contained diverse restriction enzymes sites, a common feature of prokaryotic restriction modification systems that recognize mobile genetic elements, with more restriction enzymes sites detected in the representative archaeal episome (Figure 3).

Sourmash [18] was used to examine sequence similarity for all 38 episomes carrying smBGCs (Supplementary Table S1) and indicated that all episomes contained distinct *k*-mer profiles. Clustering of our episome sequences using CD-HIT [19] at 70% sequence identity threshold did not yield any clusters. Altogether, this suggests that recovered episomes have low sequence similarity. To address the putative taxonomic origin of the identified mobile smBGCs we ran the Contig Annotation Tool (CAT; [20]). These results were largely consistent with taxonomic annotations of MAGs originally assigned by GTDB-Tk 2.1.1 and are presented in Supplementary Table S1.

## 4. Discussion

While >34,000 plasmids exist in the public plasmid database PLSDB [33] many plasmids in environmental samples remain undetected. This is because plasmid features including structure, size and gene content do not allow bioinformatic/computational tools to easily distinguish plasmid-encoded DNA from chromosomal DNA fragments (e.g., [34,35,36,37]). Further, existing bioinformatic pipelines for plasmid identification are typically designed for complete or representative genomes, and therefore, when used to analyze MAGs reconstructed from complex environmental communities (e.g., Cariaco Basin), plasmid prediction and identification can be challenging. Finally, many of the existing plasmid prediction bioinformatic tools are trained against reference databases (PLSDB) that usually contain sequences from circular, not linear, plasmids, and originate from bacteria affiliated with human pathogens, terrestrial, and freshwater taxa [16,33]. As an example, Androsiuk et al. (2023) [37] examined the marine plasmidome of the Red Sea, and they were able to conclusively identify only 7 out of 362 putative plasmids from metagenomic data. The authors pointed out the shortcomings of existing tools and databases that lead to underestimation of the marine plasmidome [37].

Using samples collected from distinct geochemical regimes within the Cariaco Basin’s water column, we investigated the presence of plasmid-borne smBGCs in MAGs affiliated with particle-associated (PA) and free-living (FL) prokaryotic communities. We identified 38 integrated plasmids in 28 MAGs (Supplementary Table S1). The small number of MAGs carrying integrated smBGCs is not a surprise considering the shortcomings of existing tools, as well as other possible ecological explanations discussed in detail below. Overall, the limited number of MAGs chosen for discussion in our Cariaco study precludes drawing conclusions about whether smBGC episomes are more commonly found in PA vs. FL prokaryotes of Cariaco Basin. DNA transfer via plasmids within PA communities is expected to be more efficient than those in the FL size fraction [38]. However, some debate exists because surface ionic charges on marine particles (as for sediments) can enhance DNA binding to particle surfaces. Consequently, transformation efficiency (uptake) of plasmid DNA may be reduced on particles [39,40]. Further, Niskin bottle-collected water samples primarily capture small, suspended particles and cells rather than rare, rapidly sinking particles [41]. Only by capturing a full representation of all sinking particles (such as collected by sediment traps) would it be possible to fully assess the role of smBGC episomes in the PA water fraction. Nonetheless, we postulate that plasmid-encoded smBGCs may present more selective advantages to particle-associated communities than to free-living counterparts for several reasons. Previous studies of Cariaco Basin showed that suspended particles are hotspots of microbial activity in the water column, and that in situ PA microbes depend on secondary metabolites to compete for nutrient resources [10,12]. Overall, particles can protect DNA from DNase degradation [42,43,44], facilitate bacterial colonization, and can sustain higher densities of active cells because they are nutrient-rich (e.g., [45]). Higher cell densities allow cells to interact more efficiently than those in the diffuse free-living fraction of the water column, likely enhancing transformation efficiency [46]. Among the prokaryotes that can acquire smBGCs through plasmids, those attached to particles in the Cariaco Basin may have a practical advantage for acquiring exogenous DNA for reasons discussed above, however, this process requires further examination.

Thirteen of the 16 identified smBGCs episomes encoded RiPPs, RiPP recognition elements (RREs), NRPs and terpene-synthezing enzymes. RREs are conserved regions that direct the post-translational modifications of RiPPs [47]. RiPPs, NRPs and terpenes are known to have antimicrobial properties which may help microorganisms to outcompete their microbial “neighbors”. Ocean deoxygenation can compress habitats for sensitive taxa and select against taxa that cannot adapt to declining oxygen [48,49]. Prokaryotes are known to retain plasmid-encoded genes in challenging environments even when these accessory genes are not immediately used [50,51]. Transfer and retention of plasmid-encoded smBGCs with antimicrobial effects could benefit those taxa in the Cariaco Basin that can acquire them, because they can increase the versatility of their hosts under dynamic and O_2_-depleted/anoxic conditions [49,52,53].

Retention of plasmid-borne genes in host genomes is strongest when the encoded genes enhance host fitness, or when these genes are rare or absent from the host’s genome [54]. Letzel et al. (2017) [55] proposed that mobile transfer of smBGCs can enable a “plug and play” type of evolution that tests relative fitness of specialized metabolites produced under stressful conditions in marine bacteria. The smBGCs we identified in this study (e.g., RiPPs, terpene synthases) are known from the literature to have diverse functions. As an example, in addition to their antimicrobial effects, RiPPs may serve as quorum sensing signaling molecules, and terpenes may participate in cell fate decision (e.g., sporulation) [56,57,58,59,60]. Metagenomic studies suggest that acquisition of smBGCs with pleiotropic functions increases the secondary metabolic diversity and contributes to host fitness (e.g., [61]). Studies of cultured bacterial strains from ODWCs can shed additional light on the function(s) of these compounds.

As previously mentioned, identification of marine smBGC episomes is likely hindered by limitations of bioinformatic pipelines and databases, as well as by the difficulties of culturing many marine prokaryotic strains (e.g., [62,63,64]). Within Cariaco Basin’s redoxcline, the majority of recovered MAGs were associated with Omnitrophota (**56**), Chloroflexota (**49**), Myxococcota (**33**), Planctomycetota (**65**), Desulfobacterota (**43**) (now classified as Thermodesulfobacteriota; [65,66]) that collectively comprised ~55% of the assembled bacterial MAGs (463 bacterial and 102 archaeal MAGs, 565 MAGs in total; see Supplementary Data and Figure 1a from [12]). Some of these phyla (e.g., Omnitrophota) are difficult to cultivate, and hence, information on their overall genomic organization is limited. However, for other phyla (e.g., Thermodesulfobacteriota) more detailed information exists as they have also been cultured under laboratory conditions and are well-characterized. In the present study, our gene annotation and antiSMASH analyses revealed MAGs encoding at least one smBGC along with genes associated with plasmid transfer. While Omnitrophota MAGs are among the 239 MAGs, it is not yet clear whether this bacterial phylum can carry plasmid-transferred smBGCs. This is because we did not identify smBGC episomes within Omnitrophota MAGs through the conservative plasmid prediction pipelines that we applied (Supplementary Table S1). Similarly, based on our gene annotation and antiSMASH analyses, Thermodesulfobacteriota MAGs were identified to encode smBGC episomes along with genes involved in transfer of plasmid DNA. This agrees with previous literature that demonstrated that sulfate reducers carry plasmids [67] and are competent to exchange mobile DNA via conjugation with other sulfate reducers (e.g., *Desulfovibio* sp.; [68,69] or even with other bacterial phyla (e.g., *E. coli* cells; [70,71] and references therein]). However, only one Thermodesulfobacteriota MAG (CarAnox_MAG_1179) was identified using the PlasFlow and PlasClass tools, which may also be due to the caveats discussed above.

While bioinformatic limitations exist, we offer a putative ecological explanation for the few MAGs identified to carry smBGC episomes in our datasets. In silico analyses of prokaryotic phyla that had at least 50 genomes deposited in GenBank [72] suggested that common taxa having high plasmid prevalence in their genomes include Pseudomonadota, Cyanobacteria, Actinobacteria, Firmicutes, and Verrucomicrobiota [72]. Overall, those bacteria with a high prevalence of plasmids are usually cosmopolitan taxa [72,73] that can be found ubiquitously in the water column (e.g., [74,75,76,77]). The presence of naturally competent bacteria is a prerequisite for exogenous DNA uptake, and in the marine environment bacteria known to be naturally competent include Alphaproteobacteria and Gammaproteobacteria [2,37,78,79,80]. In the Cariaco Basin MAG dataset that we examined here for smMBGCs, Alphaproteobacteria and Gammaproteobacteria comprised 44 out of the 463 reconstructed bacterial MAGs (9.5%), while MAGs associated with Cyanobacteria, Actinobacteria, Firmicutes and Verrucomicrobiota collectively represented 5% of the bacterial MAGs (see Supplementary Data and Figure 1a from [12]. Hence, phyla described by [72] to have a high plasmid prevalence represented only a small fraction of the bacterial MAGs recovered (14.5%; ≥75% completeness and <5% contamination). Additionally, ref. [72] suggests that lineages like Chloroflexota and Planctomycetota, constitute ~25% of our recovered MAGs, have low plasmid prevalence in their genomes. We also know from available genomic data for taxa with low plasmid prevalence (e.g., Planctomycetota), that it is mainly their terrestrial [81], and not marine representatives [82], that carry plasmids. However, this perception might change in the future when culturing methods for Planctomycetota advance. Planctomycetota are currently difficult to culture because of their innate ability to resist many antibiotics, as well as their inability to utilize common growth media used for microbiology assays [64]. Finally, we need to underscore that exchange of genetic material within prokaryotic populations in Cariaco and other ODWCs occurs also through other mechanisms, like viruses and their auxiliary metabolic genes [83,84,85]. Viral transfer of genetic material may be a more common mechanism for transfer of mobile DNA in marine ecosystems, including ODWCs and Cariaco Basin. Collectively, these putative ecological explanations along with bioinformatic caveats discussed above may explain the recovery of relatively few MAGs encoding smBGC episomes in our dataset. While our data highlight the need for improved tools and databases for detecting marine plasmids, we note there is still much to learn about the balance between viral-mediated and plasmid-mediated transfer of genetic material in ODWCs.

## 5. Conclusions

We searched 565 published MAGs recovered from water samples collected within the Cariaco Basin’s redoxcline for plasmid-encoded secondary metabolite biosynthetic gene clusters (smBGC episomes). We found 28 MAGs that contained 38 putative smBGC episomes, and we discuss 14 MAGs for which we have the strongest evidence. These 14 MAGs contain 16 smBGC episomes that encode genes for RiPPs, RiPP recognition elements (RREs), terpene synthesis and NRPs, and are affiliated with 2 archaeal phyla (Altiarchaeota, Halobacterota) and 4 bacterial phyla (Planctomycetota, Verrucomycota, Myxococcota and Pseudomonadota). These secondary metabolites may enhance the competitive advantage of free-living and particle-associated host taxa that are competent to acquire plasmid DNA in this geochemically stratified water column. However, until comparative data on smBGC episomes from other ODWCs is available, and bioinformatic tools and databases for detecting and identifying smBGC episomes improve, it is difficult to assess their evolutionary and ecological significance in the Cariaco Basin and other oxygen-depleted water column habitats.

## Figures and Tables

**Table 1:** Description of MAGs encoding putative plasmids carrying smBGCs (smBGC episomes). The water column conditions (oxycline, anoxic, euxinic) from where these MAGs were recruited are noted inside parenthesis. Asterisk (*) indicates the archaeal MAG (CarAnox_MAG_18) and bacterial MAGs (CarAnox_MAG_1576 and CarAnox_MAG_770) used as representatives for the contruction of the plasmid maps. NA: not available taxonomic assignment below class level, RPKM: Reads Per Kilobase per Million mapped reads. Repository containing all MAGs can be found at https://osf.io/usm8r/ (accessed on 13 December 2023). The DNA coverage for MAGs at the three examined biogeochemical regimes can be found at https://zenodo.org/records/10144545 (accessed on 22 July 2023). NA: Not Available.

MAG and Contig Encoding Putative Plasmids	MAG’s Completeness/ Contamination	Geochemical Regime	Transcript Read Recruitment that Plasmids Received [RPKM/Depth (m)]	smBGCs Encoded in the Plasmids	Secondary Metabolite	Phylum/Order/Family/ Genus
CarAnox_MAG_1243_4	96.88/0.93	Anoxic, Euxinic	0	RiPP	RiPP	Altiarchaeota/ SCGC-AAA252-I15/ SCGC-AAA252-I15/NA
* CarAnox_MAG_18_14	96.73/0.33	Euxinic	0.64/900	RiPP	RRE-containing	Halobacterota/ Syntrophoarchaeales/ Syntrophoarchaeaceae/ *Syntrophoarchaeum* sp.
CarAnox_MAG_1071_28	77/2.7	Anoxic, Euxinic	0	Terpene	Terpene	Verrucomicrobiota/ Kiritimatiellae/ YA12-FULL-48-11/NA
CarAnox_MAG_1075_66	91.22/2.48	Anoxic, Euxinic	0	Other	Resorcinol	Verrucomicrobiota/ Kiritimatiellae/ UBA8416/NA
CarAnox_MAG_828_1	96.28/2.39	Anoxic, Euxinic	0.03/267	RiPP hybrid	RiPP-like	Verrucomicrobiota/ Kiritimatiellae/ UBA8416/NA
CarAnox_MAG_1543_26	88.92/1.7	Euxinic	0	PKS hybrid	hglE-KS	Planctomycetota/ Phycisphaerales/ Phycisphaeraceae/NA
CarAnox_MAG_1628_60	95.33/2.4	Euxinic	0	Terpene	Terpene	Planctomycetota/ Pirellulales/ UBA1268/UBA1268 sp002694955
CarAnox_MAG_1628_81	95.33/2.4	Euxinic	0	NRPS	NRPS-like	Planctomycetota/ Pirellulales/ UBA1268/UBA1268 sp002694955
CarAnox_MAG_245_32	98.8/1.14	Euxinic	0.07/900	RiPP	RRE-containing	Planctomycetota/ Brocadiae/ SM23-32/NA
CarAnox_MAG_406_14	88.41/4.55	Anoxic, Euxinic	0	NRPS	NRPS-like	Planctomycetota/ Brocadiae/ NA/NA
CarAnox_MAG_406_39	88.41/4.55	Anoxic, euxinic	0	RiPP	RRE-containing	Planctomycetota/ Brocadiae/ NA/NA
CarAnox_MAG_879_3	86.59/3.98	Oxycline, Anoxic, Euxinic	0.05/314	Terpene	Terpene	Planctomycetota/ Phycisphaerales/ SM1A02/UBA12567
* CarAnox_MAG_1576_52	99.59/1.37	Oxycline, Anoxic, Euxinic	0.33/267	RiPP	RiPP	Pseudomonadota/ Sphingomonadales/ Sphingomonadaceae/ *Novosphingobium* sp.
CarAnox_MAG_164_53	88/2.1	Euxinic	0	RiPP	RiPP	Pseudomonadota/ Pseudomonales/ Pseudomonadaceae/ *Pseudomonas* sp.
CarAnox_MAG_782_37	88.99/0.8	Oxycline, Anoxic, Euxinic	0.09/148	RiPP	RiPP	Pseudomonadota/ Sphingomonadales/ Sphingomonadaceae/ *Erythrobacter* sp.
* CarAnox_MAG_770_69	83.87/4.52	Anoxic	0.06/200	NRPS	NRPS-like	Myxococcota/ Bradimonadia/ UBA7976/ UBA1532/NA

## Data Availability

The metagenome and metatranscriptome data generated in this study have been deposited in the NCBI database under accession code PRJNA326482. The processed metagenome-assembled genomes (in FASTA format) and biosynthetic gene cluster files (in ZIP format) are available at OSF [https://osf.io/usm8r/ accessed on 13 December 2023]. The biogeochemistry data from the CARIACO Basin Time Series Station for May to November 2014 are available through the Biological and Chemical Oceanography Data Management Office (BCO-DMO) at the Woods Hole Oceanographic Institution [https://www.bco-dmo.org/dataset/652313/data accessed on 22 July 2016]. The scripts used for these analyses are available at the following GitHub repository: https://github.com/d-mcgrath/cariaco_plasmids accessed on 6 February 2023.

## Supplementary Materials

The following supporting information can be downloaded at: https://www.mdpi.com/article/10.3390/microorganisms12050929/s1, Supplementary Table S1. Identified Cariaco MAGs containing plasmid-borne smBGCs using the two plasmid prediction tools (PlasFlow and PlasClass) (provided as excel file); Supplementary Table S2. Function and position of the encoded genes within, and outside the boarders of the plasmid-borne smBGC clusters (provided as excel file). Annotations were performed using multiple databases which are noted in the separate tabs of the excel file.
